# *Anaplasma phagocytophilum*-Related Defects in CD8, NKT, and NK Lymphocyte Cytotoxicity

**DOI:** 10.3389/fimmu.2018.00710

**Published:** 2018-04-09

**Authors:** Diana G. Scorpio, Kyoung-Seong Choi, J. Stephen Dumler

**Affiliations:** ^1^Vaccine Research Center, National Institutes of Allergy and Infectious Diseases, National Institutes of Health, Bethesda, MD, United States; ^2^College of Ecology and Environmental Science, Kyungpook National University, Sangju, South Korea; ^3^Department of Pathology, Uniformed Services University of the Health Sciences, Bethesda, MD, United States

**Keywords:** *Anaplasma phagocytophilum*, cytotoxic lymphocyte, CD107a, cytotoxicity, CD8 T cells, NKT cells, NK cells, MHCI

## Abstract

Human granulocytic anaplasmosis, caused by the tick-transmitted *Anaplasma phagocytophilum*, is not controlled by innate immunity, and induces a proinflammatory disease state with innate immune cell activation. In *A. phagocytophilum* murine infection models, hepatic injury occurs with production of IFNγ thought to be derived from NK, NKT cells, and CD8 T lymphocytes. Specific *A. phagocytophilum* ligands that drive inflammation and disease are not known, but suggest a clinical and pathophysiologic basis strikingly like macrophage activation syndrome (MAS) and hemophagocytic syndrome (HPS). We studied *in vivo* responses of NK, NKT, and CD8 T lymphocytes from infected animals for correlates of lymphocyte-mediated cytotoxicity and examined *in vitro* interactions with *A. phagocytophilum*-loaded antigen-presenting cells (APCs). Murine splenocytes were examined and found deficient in cytotoxicity as determined by CD107a expression *in vitro* for specific CTL effector subsets as determined by flow cytometry. Moreover, *A. phagocytophilum*-loaded APCs did not lead to IFNγ production among CTLs *in vitro*. These findings support the concept of impaired cytotoxicity with *A. phagocytophilum* presentation by APCs that express MHC class I and that interact with innate and adaptive immune cells with or after infection. The findings strengthen the concept of an enhanced proinflammatory phenotype, such as MAS and HPS disease states as the basis of disease and severity with *A. phagocytophilum* infection, and perhaps by other obligate intracellular bacteria.

## Introduction

Human granulocytic anaplasmosis, caused by the tick-transmitted *Anaplasma phagocytophilum*, is the third most common human vector-borne infection in the U.S., where 1% die and 7% require ICU admission (1–3). *A. phagocytophilum* is not controlled by innate immunity, but induction of a proinflammatory disease state occurs with innate immune cell activation *via* TLR2 and the inflammasome to achieve STAT1-mediated IFNγ and NF-κB-mediated proinflammatory gene transcription (4–8). As a result, infection in humans and in animal models leads to a macrophage activation syndrome (MAS) where severity is related to high serum levels of IFNγ, IL-10, IL-12, and ferritin (9–12).

In *A. phagocytophilum* murine infection models, inflammatory hepatic histopathologic injury is abrogated in *Ifng*^−/−^ and enhanced in *Il10*^−/−^ animals, confirming an important role for IFNγ in driving tissue injury (5, 6, 9, 10, 13). Key candidates for production of IFNγ include the innate immune NK and NKT cells, but also include CD8 T lymphocytes as adaptive immune responses mature. NK, NKT, and CD8 T lymphocytes react to fundamentally distinct ligands, microbe-associated molecules, or other signals (14–16). TLR2-activation implies a role for *A. phagocytophilum* lipoproteins as a ligand, and induction of the inflammasome *via* NLRC4 relates to endogenous host cell eicosanoid production following infection. However, the specific *A. phagocytophilum* ligands that drive inflammation and disease are not known (4, 17). Regardless, these observations secure an inflammatory basis for *A. phagocytophilum*-induced septic or toxic shock-like manifestations and imply that severity has its pathophysiologic basis in MAS and hemophagocytic syndrome (HPS) (11, 18).

MAS and HPS are related disorders that have either a genetic basis or an infectious trigger. Both are cytokine-driven diseases characterized by progressive fever, shock, organ failure, pancytopenia, liver dysfunction, and coagulopathy, and usually attributed to excessive IFNγ production (19, 20). Genetic forms of HPS result from mutations of genes encoding proteins involved in signaling perforin or granzyme delivery for cytolysis of target cells resulting in impaired cytotoxic lymphocyte (CTL) function, often among NK cells. Similarly, infection-associated HPS is characterized by defects in CTLs, either by NK cell lymphopenia, or NK cell defects in perforin delivery, although HPS has been observed in humans and animal models with defects in CD8 T cells as well (21). The explanation for the relentless progression with HPS and MAS is that the antigen-presenting cell (APC)-cytotoxic cell synapse through TCR–MHC class I interaction results in activation of the CTL and production of IL-12, IL-18, IL-15, and IL-2, resulting in lymphoproliferation and IFNγ generation (19, 20). IFNγ activates macrophage effector production (nitric oxide, reactive oxygen species, TNFα, phagocytosis). However, the inability of CTLs to deliver perforin to the APC presenting a cognate ligand frees the cascade from regulation, exacerbating disease due to unremitting cytokine stimulation (22).

Recent studies provide evidence that the signaling pathways generating the key functions for this response are distinct, providing a framework for understanding the dichotomy of hypercytokinemia without cytotoxicity (20, 23). To better understand the nature of MAS/HPS induced by *A. phagocytophilum* infection, we studied *in vivo* and *in vitro* responses of NK, NKT, and CD8 T lymphocytes from infected animals to determine if their interactions with *A. phagocytophilum*-loaded APCs results in delivery of cytotoxic cargo dissociated from intracellular production of IFNγ.

## Materials and Methods

### *In Vitro* Cell Line and *A. phagocytophilum* Culture

The promyelocytic leukemia HL-60 cell line (ATCC CCL-240) was used as a host for *A. phagocytophilum* growth. HL-60 cells were grown in RPMI 1640 medium (Invitrogen, USA) containing 5–10% FBS in a humidified incubator at 37°C with 5% CO_2_. Cell density was kept <5 × 10^5^ cells/mL by diluting with fresh medium every 3 days.

### Animals and Immunophenotyping

Naïve C57BL/6 mice at 6–8 weeks of age (Jackson Labs, Bar Harbor, ME, USA) were inoculated IP with 10^6^ *A. phagocytophilum* Webster strain-infected HL-60 cells. CD1d^−/−^ animals on a C57BL/6 background used in the studies of NK cells were kind gifts from Albert Bendelac (University of Chicago) and Luc Van Kaer (Vanderbilt University). Mock-infected animals were inoculated with uninfected HL-60 cells. This inoculation reproducibly generates infection by days 2–4 and up to day 14, and IFNγ production peaks between days 4 and 10 p.i. To generate immune mice for CD8 T-cell experiments, mice were treated on day 14 with doxycycline (5 mg/kg PO q12h) for 7 days followed by 7 days with no treatment to allow drug clearance; on day 28, splenocytes were harvested and tested by PCR to exclude infection (24). Mice were euthanized following CO_2_ exposure. All animal studies were reviewed and approved by the Johns Hopkins University Institutional Animal Care and Use committee. All mice were housed and cared for following the “Guide for the Care and Use of Laboratory Animals” (25).

### *Ex Vivo* Experiments to Identify Cytotoxic CTL Activation

C57BL/6 mice were inoculated i.p. with *A. phagocytophilum*-infected HL-60 cells or mock-infected by uninfected HL-60 cells as described above. For evaluation of *in vivo* activation of cytotoxicity for CD8 T lymphocytes and NKT cells, splenocytes were harvested at 4 h, and days 4, 7, 10, and 14 p.i., and processed for flow cytometry. Spleens from individual mice were minced to obtain single-cell suspensions, washed and erythrocytes lysed in a hypotonic salt solution, and then resuspended in RPMI 1640 medium with 10% FBS and 1× penicillin/streptomycin. Single-cell suspensions were stained for 20 min on ice using antibodies to CD107a (BD Biosciences) and (i) for CD8 CTLs using anti-CD3ε (BD Biosciences) and R-PE-conjugated anti-CD8a (Ly-2) (BD Biosciences), or (ii) for NKT cells using α-galactosylceramide (αGC)-loaded CD1d:Ig dimers (Mouse DimerX, BD Biosciences). All studies also used isotype-matched control antibodies (BD Biosciences). The stained cells were washed twice with phosphate-buffered saline containing 0.5% bovine serum albumin (Sigma, St. Louis, MO, USA) and 0.02% NaN_3_, washed again, and fixed. Cells were examined by multicolor flow cytometry comparing the proportion of splenocytes expressing each marker among infected and uninfected animals. Data were analyzed with FlowJo software (Tree Star, Ashland, OR, USA), and gates and fluorescent cutoffs were set based on isotype-matched control antibodies. Stained cells were initially gated to identify lymphocyte populations which were then quantified by flow cytometry per quadrant for 2 by 2 fluorescent cell markers.

### Cell Preparations for *In Vitro* Determination of CTL Cytotoxicity by CD107a Expression

For these experiments, donor mice were used as source of splenic CD8 T, NKT, or NK lymphocytes; immune CD8 splenic T lymphocytes were harvested from immune animals prepared as described above and prepared as single-cell suspensions (26). Splenocytes were also obtained as sources of dendritic cells (DCs) and APCs, including from C57BL/6 and CD1d^−/−^ mice as appropriate. DCs (Miltenyi Mouse DC Isolation, Auburn, CA, USA) or APCs from C57BL/6 mouse spleens (27) were loaded with viable cell-free *A. phagocytophilum* for 24–48 h; cell-free bacteria were removed by centrifugation and washing. Splenocytes that included unfractionated CTLs from naïve and immune animals were added to 96-well plates with *A. phagocytophilum*-loaded (or mock-loaded) DCs in effector: target ratios from 0.1:1 to 5:1 and anti-CD107a-FITC or isotype control antibodies (BD Biosciences) for 24–48 h. After multicolor flow cytometry to identify CD3^+^/CD8^+^, CD49b^+^ (in CD1d^−/−^ animals to preclude NKT cell responses), or CD3^+^/Vα14^+^ lymphocytes that also express the cytotoxicity marker CD107a, groups were compared to controls to demonstrate intact CTL functions.

Positive controls included concanavalin A (ConA), phorbol-12-myristate-13-acetate (PMA; CD8 T and NKT lymphocytes), α-galactosylceramide (αGalCer; NKT lymphocytes), or N-α-Palmitoyl-S-[2,3-bis(palmitoyloxy)-(2RS)-propyl]-l-cysteine (Pam_3_Cys; NK cells); PMA/ionomycin were included in assays as positive control for intracellular IFNγ production, and negative controls received diluent vehicle only. For NKT analyses, controls included mock-loaded and αGalCer-loaded DCs, as well as stimulation by PMA/ionomycin. For NK cell analyses, controls included mock-loaded DCs, the TLR2 agonist Pam_3_Cys, and PMA/ionomycin. Splenocyte cultures from naïve or immune animals were incubated with DCs for 1 h at 37°C, and then received brefeldin A to facilitate intracellular IFNγ detection. Cells were washed, saponin permeabilized, and stained with fluorescent anti-IFNγ, anti-CD3, anti-CD8, anti-CD49b (pan-NK cell marker), anti-Vα14 (NKT cell marker) or isotype-matched control antibodies, washed again, and fixed. The cells of interest were identified by multicolor flow cytometry, as previously described (8, 26). Expression of CD107a and intracellular IFNγ were examined and reported as a percentage of total cells analyzed for the subset examined (CD3^+^/CD8^+^, CD3^+^/Vα14^+^, CD3^−^/CD49b^+^). This allowed simultaneous evaluation of three cytotoxic cell subsets and their independent expression of cytokines and activation for cytotoxicity. Experiments were conducted in at least triplicate, and final data were also pooled for more detailed examination.

### Statistical Analysis

Comparisons were performed across all groups within each CTL experiment. Expression of CD107a or IFNγ was examined and if not normally distributed, results were ranked (using percent rank) and examined by two-tailed Mann–Whitney tests with an α-value of 0.05. Results were analyzed individually in at least three repeated experiments for each condition, and the final ranked results were pooled for an overall statistical analysis using the same methods. Results reported reflect the relative cytotoxicity (CD107a expression) and intracellular IFNγ production comparing responses of CTLs to *A. phagocytophilum*-loaded or mock-loaded DCs. Results were considered significant if the *p*-value was less than 0.05.

## Results

### *A. phagocytophilum* Infection Suppresses CD8 T Lymphocyte and NKT Lymphocyte Cytotoxicity *In Vivo*

**Figure 1:**
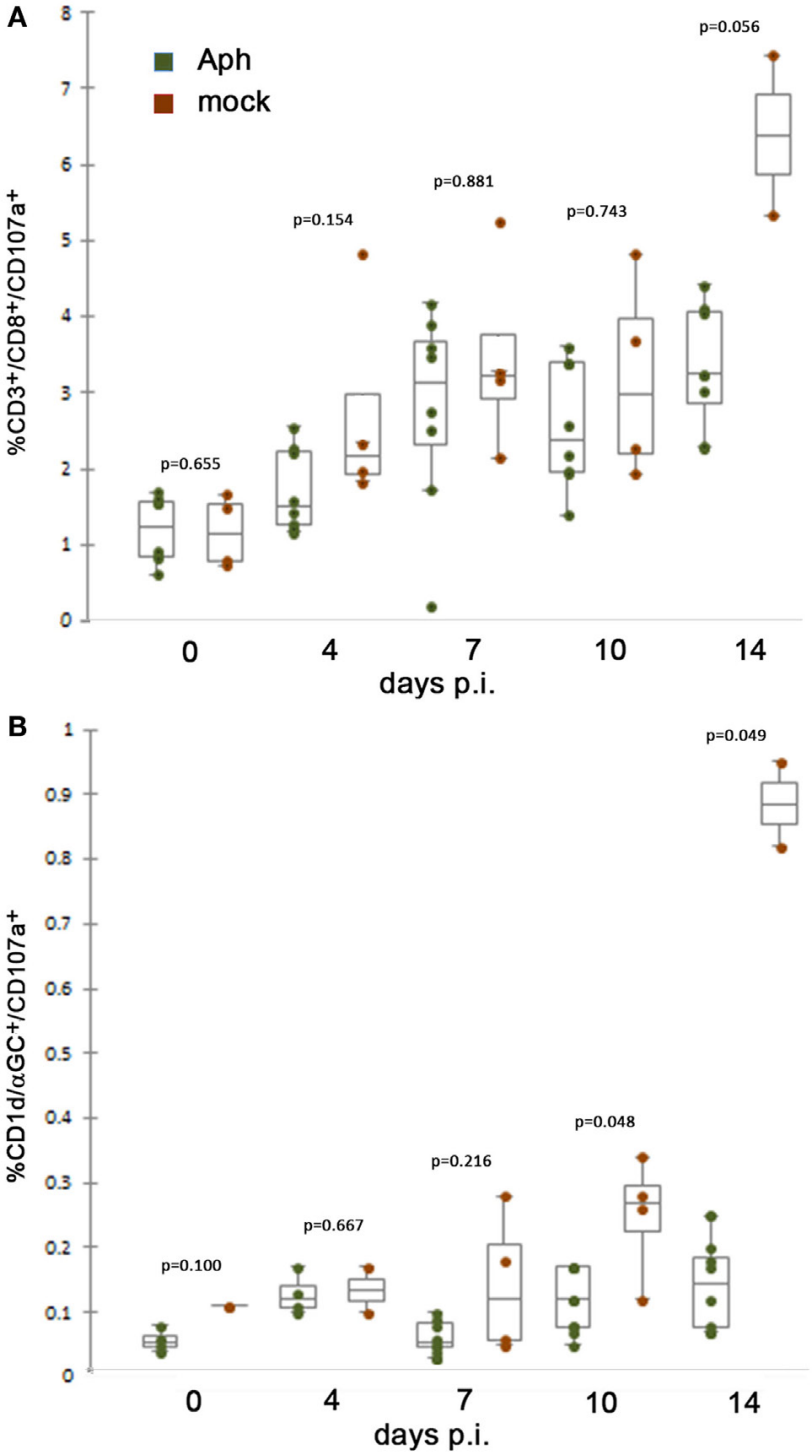
At 10 days or later p.i., *in vivo* splenic CD8 and NKT cytotoxic lymphocyte CD107a expression is lower after infection by *A. phagocytophilum* in HL-60 cells compared to mock infection by uninfected HL-60 cells. **(A)** Splenic CD8 T lymphocytes from mock-infected (uninfected HL-60 cells) animals displayed mobilized cytotoxic responses as measured by surface CD107a expression at higher levels than with *A. phagocytophilum*-infected HL-60 at 14 days p.i. **(B)** Splenic NKT cells from infected animals demonstrate similar lack of significant cytotoxicity as measured by expression of CD107a compared to animals mock infected (uninfected HL-60 cells) at days 10 and 14 p.i. Individual points represent values from individual animals; box and whisker plots show median, first and second quartiles, and maximum and minimum values for each group. *p* Values are displayed comparing groups on individual days. Aph, *Anaplasma phagocytophilum*.

We used expression of CD107a as a surrogate measure of degranulation and cytotoxicity after exposure to APCs presenting appropriate peptides or NKT-target glycolipids. When splenocytes were gated to identify the proportions of CD3^+^/CD8^+^ T lymphocytes also expressing CD107a, there was an increase from days 0 to 14 for both infected and mock-infected mice. For each of days 0–10, expression of CD107a on CD3^+^/CD8^+^ T cells from uninfected HL-60 cell (mock) controls was higher than that observed for cells from infected animals, although the results were not significant. However, on day 14 p.i., the increase in cytotoxicity (CD107a expression) among cells from mock-infected animals was more exaggerated but not significantly higher (*p* = 0.056, Mann–Whitney test) than observed among cells from infected animals (Figure 1A). Similarly, CD107a expression on NKT cells was generally unchanged among infected animals over the 14-day experiment, whereas control mice who received uninfected HL-60 cells, a xenogeneic cell line anticipated to stimulate cytotoxic responses, demonstrated a slow increase in CD8 and NK T lymphocyte cytotoxicity (CD107a expression) as early as day 7 p.i., peaking at day 14 at the experiment’s conclusion by contrast, demonstrating significant suppression of responses in cells from infected animals at days 10 and 14 (*p* = 0.048 and 0.049, respectively; Mann–Whitney test) (Figure 1B).

### *In Vitro* Suppression of CTL Expression of CD107a With Stimulation by *A. phagocytophilum*-Loaded DCs

**Figure 2:**
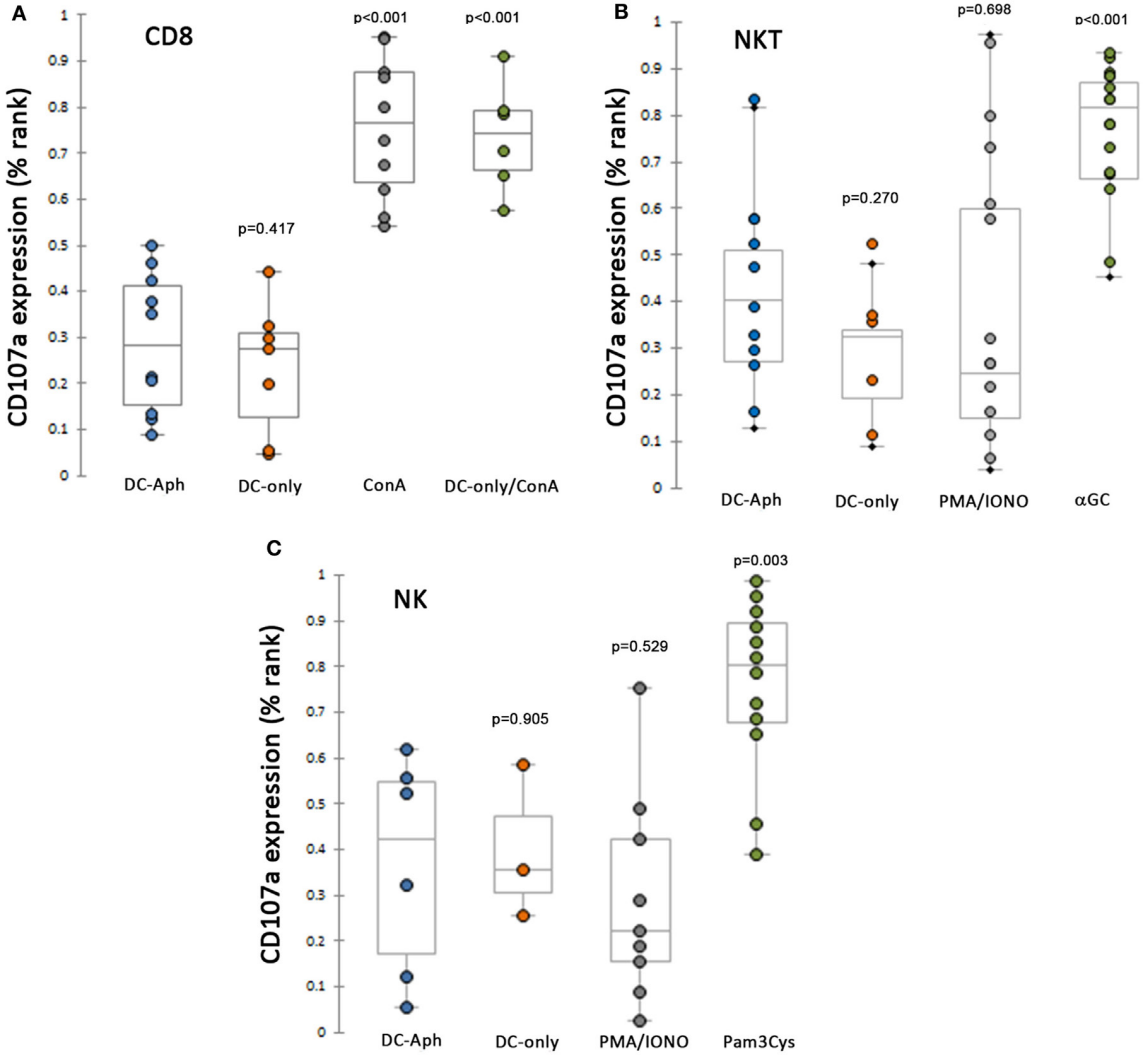
CD8 immune T, NKT, and NK lymphocytes are suppressed from cytotoxicity (CD107a expression) when exposed to *A. phagocytophilum*-loaded dendritic cells (DCs). **(A)** Splenic CD8 T lymphocytes from immune animals are suppressed from expressing CD107a to a level observed with mock-loaded DCs, and significantly lower than when cells were stimulated by ConA. **(B)** As above, splenic NKT cells are unable to generate CD107a as a cytotoxic reporter when stimulated by *A. phagocytophilum*-loaded DCs more than mock stimulation, despite effective cytotoxic responses observed to control stimulus αGalCer-loaded DCs. **(C)** NK cells are suppressed from expression of CD107a under the same circumstances, except for the use of the control stimulus Pam_3_Cys, a TLR2 agonist. DC-only, mock-loaded DCs; DC-Aph, *A. phagocytophilum*-loaded DCs; ConA, concanavalin A; αGC, α-galactosylceramide; PMA/IONO, phorbol-12-myristate-13-acetate/ionomycin C; Pam_3_Cys, N-α-Palmitoyl-S-[2,3-bis(palmitoyloxy)-(2RS)-propyl]-l-cysteine. Variable results are shown for the use of PMA/ionomycin in generated cytotoxic responses. Responses are measured as the proportion of each gated cell population expressing CD107a divided by the total number of the gated cell population. Because the values were not normally distributed, the proportions were ranked by percentage and tested using Mann–Whitney tests for non-parametric significance, with a two-sided α = 0.05. *p* Values are shown compared to DC-Aph for each condition. Aph, *Anaplasma phagocytophilum*.

Because the *in vivo* experiments suggested suppression of CTL responses by *A. phagocytophilum* infection, splenocytes, as sources of NK, NKT, and CD8 T lymphocytes from immune or naïve animals, were exposed to DCs preloaded with *A. phagocytophilum* (Figure 2). Compared to responses generated with exposure to mock-loaded DCs, CD3^+^/CD8^+^ splenic lymphocytes from immune animals stimulated by *A. phagocytophilum*-loaded DCs did not generate additional CD107a expression (Figure 2A), but demonstrated a suppressed response as compared to immune splenic CD3^+^/CD8^+^ lymphocytes exposed to ConA-stimulation (*p* < 0.001). CD107a expression to ionomycin was variable, but this treatment is established to render NK cells hyporesponsive (28). Thus, the CD3^+^/CD8^+^ immune lymphocytes retained functional capacity to degranulate but were suppressed in the presence of *A. phagocytophilum*-loaded DCs.

Similarly, splenocytes from naïve mice and from CD1d^−/−^ mice were used to assess cytotoxicity responses to *A. phagocytophilum*-loaded DCs in NKT (Figure 2B) and NK (Figure 2C) cells, respectively. For both NKT and NK cells among the splenocyte populations, cytotoxicity responses (CD107a expression) were significantly higher than negative control mock-loaded DCs for positive controls [αGalCer-loaded DCs (*p* < 0.001) or Pam_3_Cys (*p* = 0.003)]. By contrast, compared to the cytotoxicity responses of NKT and NK cells when exposed to positive control stimulants, exposure to *A. phagocytophilum*-loaded DCs suppressed cytotoxicity to levels not different than negative control mock-loaded DCs.

### Intracellular IFNγ Production Is Impaired in Splenic CTLs

**Figure 3:**
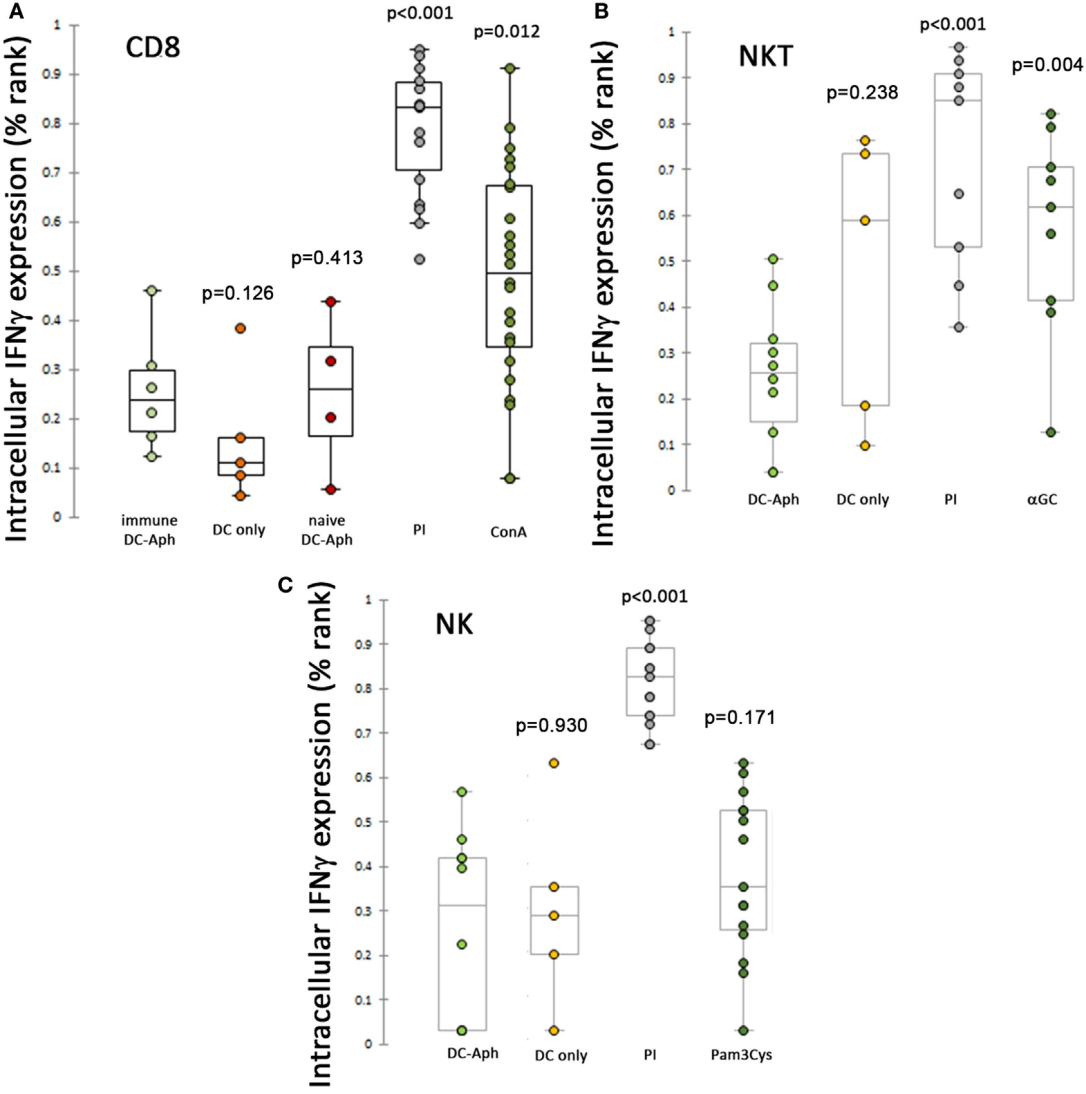
Suppression of intracellular IFNγ production after stimulation of splenic cytotoxic lymphocytes (CTLs) by *A. phagocytophilum*-loaded dendritic cells (DCs). Intracellular production of IFNγ was measured as described in Figure 2 and in the Section “Materials and Methods.” Immune CD8 T **(A)**, NKT **(B)**, and NK **(C)** lymphocytes were suppressed from *A. phagocytophilum*-induced IFNγ expression as compared with positive control stimuli PMA/Ionomycin (all CTLs; **A–C**), ConA (CD8 T lymphocytes; **A**), and αGalCer (NKT lymphocytes; **B**). *P* values are generated from ranked data (as described in the Section “Materials and Methods” and Figure 2) and are displayed over each variable compared to the cells exposed to *A. phagocytophilum*-loaded DCs (DC-Aph). Aph, *Anaplasma phagocytophilum*.

Given that *A. phagocytophilum*-loaded DCs suppressed cytotoxicity responses as measured by CD107a expression on splenic CTLs, we next examined whether these cells also were suppressed for the production of IFNγ, by examining the intracellular production of this key macrophage-activating cytokine (Figure 3). Surprisingly, *A. phagocytophilum*-immune CD8 T lymphocyte (Figure 3A), NKT cell (Figure 3B), and NK cell (Figure 3C) expression of IFNγ from splenocytes was suppressed when exposed to *A. phagocytophilum-*loaded DCs as compared with stimulation by PMA/ionomycin (CD8 T cells, NKT cells, and NK cells all *p*<0.001), ConA [immune (*p* = 0.012) and naïve CD8 T cells (*p* = 0.413)], and αGalCer-loaded DCs [NKT cells (*p* = 0.004)]. Pam_3_Cys proved to be a poor stimulant for IFNγ production in NK cells. Of note, immune CD8 T lymphocytes exposed to *A. phagocytophilum*-loaded DCs did not stimulate IFNγ production more than either mock-loaded DCs or naïve CD8 T lymphocytes exposed to *A. phagocytophilum*-loaded DCs, and neither NK nor NKT cells generated more IFNγ when exposed to *A. phagocytophilum*-loaded DCs (wild type or CD1d^−/−^ for NK cells) than for mock-loaded DCs. In summary, immune CD8 T, NKT, nor NK lymphocytes were unable to elicit IFNγ intracellular production when exposed to *A. phagocytophilum*-loaded DCs, despite the ready capacity of these cells to produce IFNγ with positive control stimulants.

## Discussion

Obligate intracellular bacteria such as *A. phagocytophilum* use their host cells as part of an extended environment and have evolved a capacity to interact with and manipulate host cells to improve microbial fitness, sometimes at the cost of damage to the host in the form of disease (18, 29, 30). Studies of *A. phagocytophilum*, therefore, focus on two important aspects of the unique bacterium, how it survives and propagates within the key host defense cell, the neutrophil, and how it causes disease, which increasingly is documented to involve inflammatory and immune induction (18, 31–33). Much has been studied regarding mechanisms by which the immune response resolves *A. phagocytophilum* infection, largely predicated on the development of CD4 T lymphocyte responses (5, 34). During investigations of immune pathways involved in resolving infection, it was discovered that although many innate and adaptive immune pathways are activated, few impact microbial burden (5). Of great interest was the discrepancy between bacterial load and inflammatory tissue injury or disease manifestations in humans and animal models (9, 10). It has increasingly become clear that infection of the neutrophil likely contributes to disease by induction of proinflammatory responses and lack of antimicrobial killing (18, 33), but that key protective mechanisms are initiated *via* macrophages and other APCs that do not sustain infection, but contribute to the proinflammatory disease process and severity of infection (17, 35–37). In fact, aspects of granulocytic anaplasmosis in humans and animals mimic MASs and HPSs (10–12). We sought to discern whether *A. phagocytophilum* induces MAS or HPS driven by defective CTL delivery of perforin and granzyme while promoting hypercytokinemia primarily focused on IFNγ to further activate macrophages. Thus, we designed *in vivo* pilot studies and *in vitro* studies to examine the roles of immune CD8 T lymphocytes, NK cells, and NKT lymphocytes to interrogate both cytotoxicity responses and promotion of IFNγ production.

The findings from this investigation support the concept of impaired cytotoxicity of innate and adaptive immune CTLs that are, thus, unable to exert homeostatic control of APCs following antigen processing and MHC class I expression to these CTLs. While IFNγ is readily detected during *in vivo* infections, the relative lack of its expression in CTLs after exposure to APCs that should otherwise present microbial targets for activation suggests that it originates from other sources and that the functional defect could instead reside within the APC. While these data do not support the classical paradigm toward development of MAS (19), the ongoing production of IFNγ by other cells coupled with the lack of effective feedback cytotoxicity by classical CTLs would be equivalent.

*Anaplasma phagocytophilum* can stimulate proinflammatory responses in macrophages *in vitro* through TLR2, presumably *via* a lipoprotein (4, 38). However, the recognition that intracellular pathogens are also sensed and controlled *via* intracellular pattern recognition receptors has triggered a line of investigations into nucleotide-binding domain and leucine-rich repeat containing proteins (NLRs) (37, 39). In fact, recent studies showed that *A. phagocytophilum* activates the inflammasome in macrophages by a novel process that involves NLRC4 and production of endogenous eicosanoids (17). Moreover, studies of inflammasome activation by this and other obligate intracellular bacteria that lack classical pathogen-associated molecular patterns (e.g., type III secretion system components or cell wall products) are now just being initiated.

*Anaplasma phagocytophilum* does not productively infect macrophages or presumably other APCs that could be involved in CTL engagement and activation of cytotoxicity (40). Therefore, how could its presence in APCs lead to dysfunctional recognition by immune CD8 T cells, NKT cells, and NK cells? The bacterium’s lifestyle is obligatory intracellular, and it likely stimulates APCs in delivery of secreted effector proteins or molecules *via* type IV secretion or other methods to translocate such products into the cell’s cytosol for MHC class I processing (41). By contrast, its ability to engage surfaces of myeloid and monocytic cells suggests effective endocytosis but ineffective remodeling of the newly formed vacuole for prolonged survival within monocyte-differentiated cells (32). It is likely that these vacuoles fuse with the degradative complexes that precede antigen processing and presentation, typically the domain of MHC class II presentation. Given the lack of the ability to generate effective cytotoxicity across three major CTL types (CD8, NKT, and NK cells) suggest a defect in processing for MHC class I presentation within APCs. Permutations for the MHC class I processing route could occur at delivery into or with proteasome degradation within the cytosol, delivery through transporter associated with antigen processing protein into the endoplasmic reticulum, proteolytic processing within the endoplasmic reticulum, loading of cargo onto MHC class I molecules, or delivery of the loaded MHC class I to the cell surface. While there are potential interactions that could be experimentally tested (32, 42, 43), little data currently exist to support most of these potential points of subversion.

Could *A. phagocytophilum* products derived *via* the processing for presentation by APCs impact its ability to present and activate CTLs? Among annotated NLRs, recent studies performed implicate NLRC5 as the key regulator of MHC class I gene expression (44–46). Regulation of NLRC5 expression is unclear, but likely linked in part to stimulation by type I and II interferons (47), Poly I:C (a mimic of double stranded RNA) (48), and possibly by bacterial porins, of which *A. phagocytophilum* expresses large quantities (49). Is it plausible that *A. phagocytophilum* impairs MHC class I expression *via* NLRC5, resulting in the observed failure to activate cytotoxicity and IFNγ generation in *A. phagocytophilum*-specific responses? We recently completed RNAseq transcriptional profiling of *A. phagocytophilum* infection in ATRA-differentiated HL-60 promyelocytic leukemia cells and examined splice variant transcripts (unpublished data). Although the studies were conducted in a cell differentiated toward myeloid maturity, within the transcriptome were identified 17 distinct alternative isoforms of NLRC5, all but one which were not differentially regulated by the infection; however, one nonsense-mediated decay isoform, *NLRC5*-010, was expressed at least ninefold greater with infection. Nonsense-mediated decay forms are now recognized as regulators of transcription in cell differentiation, in response to stress, and in development of disease (50). We suggest that additional studies to investigate whether this isoform transcript could affect NLRC5 and MHC class I expression should be conducted.

The results of this study will drive future investigations into the cellular and molecular mechanisms for the CTL functional dissociation of MASs. These data also provide another avenue to explore acquired or infectious MASs unrelated to this pathogen through the conduct of *in vivo* cytotoxicity studies. Such investigations should confirm *in vitro* studies and illustrate which cells possess *in vivo* cytotoxicity, and whether impaired cytotoxicity leads to an enhanced proinflammatory phenotype which would recapitulate MAS and HPS disease states, and whether approaches directed at MHC class I expression might be fruitful avenues for control of disease manifestations.

## Ethics Statement

All animal studies were reviewed and approved by the Johns Hopkins University Institutional Animal Care and Use committee. All mice were housed and cared for following the “Guide for the Care and Use of Laboratory Animals” (25).

## Author Contributions

DS helped conceive the work, conduct experimentation, interpret results, and write the manuscript. KSC helped to conceive the work, conducted most of the research, interpreted results, and helped to edit the manuscript. JD helped conceived the work, interpret the results, and write the manuscript.

## Disclaimer

The opinions expressed herein are those of the author(s) and are not necessarily representative of those of the Uniformed Services University of the Health Sciences (USUHS), the Department of Defense (DOD); or, the United States Army, Navy, or Air Force.

## Conflict of Interest Statement

The research was conducted in the absence of any commercial or financial relationships that could be construed as a potential conflict of interest.
